# Comprehensive analysis reveals dual biological function roles of EpCAM in kidney renal clear cell carcinoma

**DOI:** 10.1016/j.heliyon.2023.e23505

**Published:** 2023-12-14

**Authors:** Mei Chen, Yuanhui Gao, Hui Cao, Zhenting Wang, Shufang Zhang

**Affiliations:** a Central Laboratory, Haikou Affiliated Hospital of Central South University Xiangya School of Medicine, Haikou, 570208, China; b Urology, Haikou Affiliated Hospital of Central South University Xiangya School of Medicine, Haikou, 570208, China

**Keywords:** EpCAM, Pan-cancer, Kidney renal clear cell carcinoma, Prognosis, Proliferation, Metastasis, Immunity

## Abstract

**Background:**

Epithelial cell adhesion molecule (EpCAM), a well-established marker for circulating tumor cells, plays a crucial role in the complex process of cancer metastasis. The primary objective of this investigation is to study EpCAM expression in pan-cancer and elucidate its significance in the context of kidney renal clear cell carcinoma (KIRC).

**Methods:**

Data obtained from the public database was harnessed for the comprehensive assessment of the EpCAM expression levels and prognostic and clinicopathological correlations in thirty-three types of cancer. EpCAM was validated in our own KIRC sequencing and immunohistochemical cohorts. Subsequently, an in-depth exploration was conducted to scrutinize the interrelationship between EpCAM and various facets, including immune cells, immune checkpoints, and chemotherapy drugs. We employed Cox regression analysis to identify prognostic immunomodulators associated with EpCAM, which were subsequently utilized in the development of a prognostic model. The model was validated in our own clinical cohort and public datasets, and compared with 137 published models. The role of EpCAM in KIRC was explored by biological function experiments in vitro.

**Results:**

While EpCAM exhibited pronounced overexpression across a wide spectrum of cancer types, a notable reduction was observed in KIRC tissues. As grade increased, EpCAM expression decreased. EpCAM expression decreased in patients without metastasis. EpCAM mRNA and protein levels were used as independent, favorable prognostic factors in patients with KIRC in our own cohort. The expression of EpCAM exhibited strong associations with immune-related pathways, demonstrating an inverse correlation with the majority of immune cell types. Immune checkpoint inhibitors exert better therapeutic effects on patients with low EpCAM expression. In addition, EpCAM can be used as a drug resistance indicator and guide the clinical medication of patients with KIRC. A robust model, which had good predictive accuracy and applicability, showed significant superiority over other models. Importantly, EpCAM played the dual roles of promoting proliferation and resisting metastasis in KIRC.

**Conclusion:**

In the context of KIRC, EpCAM assumes a surprising dual role, where it not only facilitates cell proliferation but also exerts resistance against the metastatic process. EpCAM serves as a standalone prognostic marker for patients with KIRC, and related models can also effectively predict prognosis. These discoveries offer novel perspectives on the functional significance of EpCAM in the context of KIRC.

## 1 Introduction

Cell adhesion molecules (CAMs) represent a category of biomolecules situated on the cell surface, mediating cellular interactions both in the context of cell-cell and cell-extracellular matrix connections. CAMs on the surface of tumor cells are closely related to cancer progression, and activate tumor signaling pathways by binding with ligands such as cells or the extracellular matrix. Epithelial cell adhesion molecule (EpCAM) constitutes a member of the cell adhesion molecules (CAMs) and manifests itself as a transmembrane glycoprotein situated on the basolateral membrane of epithelial cells.

EpCAM serves as a well-established marker for the detection of circulating tumor cells [[1], [2], [3], [4]] and the identification of cancer stem cells [5]. In addition, EpCAM serves as an invaluable biomarker in the realm of oncology, offering diagnostic capabilities for endometrial cancer [6] and lymph node metastasis detection in early-stage gastric cancer patients [7]. Patients diagnosed with esophageal adenocarcinoma may benefit from utilizing EpCAM as a promising therapeutic target [8]. Unexpectedly, EpCAM plays dual roles in endometrial cancer, promoting cell proliferation and inhibiting invasion [9]. As a tumor antigen, EpCAM can be used as an immunotherapeutic target for patients with cancer. Its neutralizing antibody, EpAb2-6, can reduce the expression of PD-L1 protein and increase CD8^+^ T cell toxicity [10]. The combination of celecoxib with interleukin-activated T lymphocytes exhibits a potential to surmount drug resistance in EpCAM-positive triple-negative breast cancer cells [11]. Monoclonal antibodies targeting EpCAM have found applications in the treatment of gastrointestinal malignancies as well as oral squamous cell carcinoma in patient populations [12,13]. Kidney renal clear cell carcinoma (KIRC) constitutes a prevailing malignancy within the urinary system, representing approximately 80 % of all renal cell carcinoma instances [14]. At present, the involvement of EpCAM in KIRC and its connection with immunity remains to be fully elucidated.

In the course of our investigation, we conducted a comprehensive analysis encompassing thirty-three different tumor types to scrutinize the expression patterns of EpCAM. Our assessment extended to exploring its clinical implications and prognostic value. To substantiate our findings, we performed validation using both our own KIRC sequencing and immunohistochemical cohorts. Subsequently, we investigated the interrelation of EpCAM with immune cells, immune checkpoints, and chemotherapy drugs. Based on EpCAM related immune regulator gene, a robust prognosis model was constructed and verified in our clinical cohort and compared with 137 published models. Finally, experimental investigations were conducted to elucidate the contribution of EpCAM to the progression of KIRC.

## 2 Materials and methods

### **2.1** Source of data

We procured transcriptomic data (fragments per kilobase million) for a comprehensive range of thirty-three cancer types through the University of California Santa Cruz Xena database (https://xena.ucsc.edu/). Additionally, we obtained clinical and survival data for the patients from The Cancer Genome Atlas (TCGA) database, which is accessible at https://portal.gdc.cancer.gov/. Utilizing data retrieved from the Oncomine database at https://www.oncomine.org/, we performed an investigation into EpCAM expression across a range of tumor types. We acquired data from the Gene Expression Omnibus (GEO) dataset GSE22541 [15], available at https://www.ncbi.nlm.nih.gov/geo/. This dataset encompassed expression profiles of 24 KIRC specimens and an additional 24 samples from KIRC metastases. The platform file designated as GPL570 is utilized for this study. We convert the probes into gene IDs. In cases where multiple probes are associated with a single gene, the gene's expression is determined by calculating the average expression of these probes. We retrieved matrix files containing transcriptome profiles and prognostic data of 101 patients with KIRC from the Arrayexpress database (https://www.ebi.ac.uk/biostudies/arrayexpress) under the accession code E-MTAB-1980 [16]. The probe ID in the matrix file is converted to a Gene bank ID and annotated as a Gene symbol. The EpCAM expression levels were sourced from TIMER accessible at http://timer.cistrome.org/.

### 2.2 Gene set enrichment analysis (GSEA)

We conducted data analysis employing GSEA 4.1.0 software to pinpoint the pathways that exhibited enrichment within both high and low expression cohorts. Our selection criteria for screening entailed a significance threshold of *P* < 0.05 and a false discovery rate (FDR) of less than 0.25. The comprehensive procedure is detailed in our prior investigation for reference [17].

### 2.3 An investigation into the interrelationship between EpCAM and immune cell populations

To appraise the immune cell composition within each sample, we applied the CIBERSORT algorithm. Following this, we performed single-sample gene set enrichment analysis (ssGSEA) to scrutinize the immune cell scores and functions in the high and low EpCAM expression cohorts. Our evaluation of immune checkpoint expression levels in these groups was carried out using the “limma” package.

### 2.4 Development of a predictive model involving EpCAM immune

We procured a selection of twelve immunoinhibitors and twenty-three immunostimulators from the TISIDB database, available at http://cis.hku.hk/TISIDB/.

We utilized univariate Cox regression analysis to identify prognostic genes, while immune-related genes employed in constructing the prognostic model were determined via multivariate Cox regression analysis. Risk score = Coefficient gene_1_ × expression of gene_1_ + Coefficient gene_2_ × expression of gene_2_ + … + Coefficient gene_n_ × expression of gene_n_.

### 2.5 Sensitivity analysis of EpCAM and anticancer drugs

The data extracted from the GDSC database (https://www.cancerrxgene.org/) were employed to assess the association between EpCAM expression and the drug's half-maximal inhibitory concentration (IC50).

### 2.6 Clinical sample collection

The cancer tissues of eighty patients with KIRC and thirty paired normal tissues from the Haikou Affiliated Hospital of Central South University Xiangya School of Medicine were collected. Prior to sample collection, we obtained approval from the hospital's ethics committee and secured informed consent from the participating patients (No. 2022-038).

### 2.7 Quantitative real-time PCR

The total RNA of the samples with Trizol (Vazyme, Nanjing, China) was extracted and then reversed to cDNA. We carried out PCR employing SYBR qPCR Master Mix (Vazyme) along with the Applied Biosystems QuantStudio 5 Real-Time PCR system. The primer sequences specific to our experimental design have been meticulously detailed in Supplementary Table S1.

### 2.8 Transcriptome sequencing

In our experimental procedure, we initially measured both the RNA quantity and its integrity for each individual sample. Subsequently, we performed polyadenylated (Poly A) RNA purification using Dynabeads Oligo (dT)25–61005 (Thermo Fisher, USA) with two successive purification steps. Following this, we fragmented the Poly A RNA into smaller segments using the Magnesium RNA Fragmentation Module (NEB, USA). Finally, the sequencing of the library was executed using the Illumina Novaseq™ 6000 platform (LC-Bio Technology Co., Ltd., Hangzhou, China).

### 2.9 Immunohistochemistry (IHC)

In this investigation, we included a total of 106 paraffin-embedded tumor tissues. In addition, we paired 63 paraffin-embedded normal tissues for (IHC). The paraffin sections were subjected to dewaxing, and we performed antigen repair following established protocols. Following the blockade of endogenous peroxidase activity, a 3 % solution of bovine serum albumin was applied and allowed to incubate at room temperature for 30 min. Subsequently, the anti-EpCAM antibody (ab223582, Abcam, England, at a dilution of 1:500) was applied and incubated overnight. Next, a secondary antibody (GB23303, Servicebio, Wuhan, China) was introduced at a dilution of 1:200. Color development was achieved through the addition of diaminobenzidine (DAB).

### 2.10 Cell biological function experiment

Cells (786-0 and 769-P) were infected with the EpCAM overexpression lentivirus. For the cell counting kit 8 (CCK-8) assay, we conducted experiments involving 786-0 (at a density of 1000 cells per well) and 769-P (at a density of 3000 cells per well) by seeding them in 96-well plates. Optical density measurements were taken at time intervals of 0, 1, 2, and 3 days. For the colony-formation assay, we utilized 6-well plates to culture 786-0 (1000 cells per well) and 769-P (2000 cells per well) over a period of 10 days. Subsequently, crystal violet staining was applied to assess colony formation. In the context of the wound-healing assay, we utilized 6-well plates to seed 786-0 cells at a concentration of 5 × 10^5^ cells per well, while 769-P cells were seeded at a concentration of 6 × 10^5^ cells per well. Once cell confluence was achieved on the second day, scratches were generated using a 100 μl pipette tip. Following two rounds of washing with PBS, serum-free medium was introduced, and images were captured using a live cell workstation. To conduct the transwell migration assay, cells were loaded into the upper chamber, and serum-free medium was introduced into the lower chamber. Following a 24-h incubation period, cell staining with 0.1 % crystal violet was performed to evaluate migration. For the invasion experiment, Matrigel was exclusively placed in the upper chamber, with all other steps remaining consistent with the migration assay. The detailed procedure of the above experiment is referred to our previous study [18].

### 2.11 Statistical processing

Statistical analyses were executed using the R software version 4.1.2. To compare EpCAM expression levels in cancer and normal tissues, the Wilcoxon test was utilized. The prognostic significance of EpCAM was assessed using Kaplan-Meier (K-M) survival analysis. Additionally, we evaluated the predictive accuracy of EpCAM by constructing a receiver operating characteristic (ROC) curve. A significance level of *P* < 0.05 was the threshold applied to establish statistical distinctions.

## 3 Results

### 3.1 EpCAM expression and correlation analysis of clinicopathological variables in pan-cancer

**Fig. 1:**
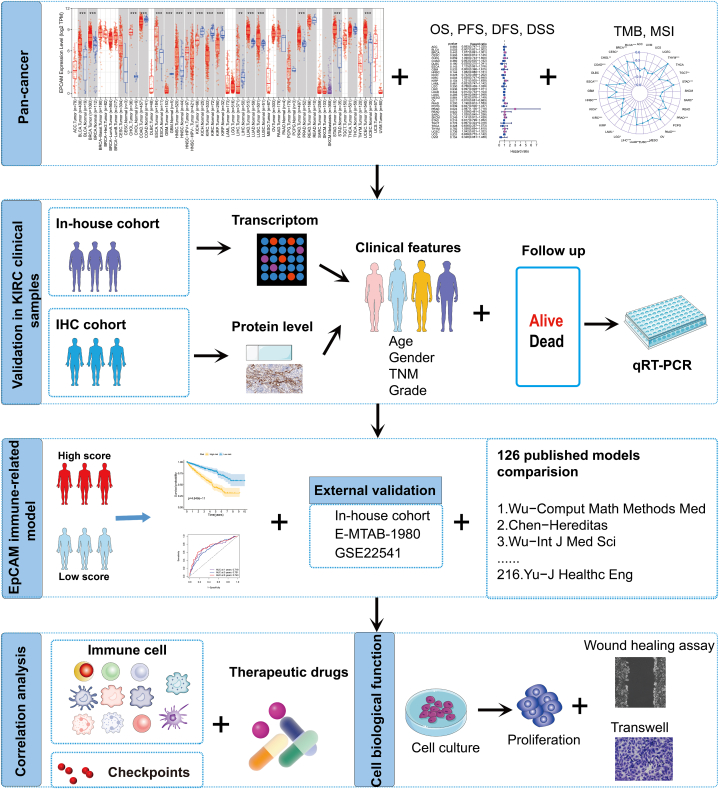
The flow chart of this study. A comprehensive investigation was conducted into the expression profile and prognostic relevance of EpCAM across diverse cancer types. Subsequently, our findings were validated using clinical samples from KIRC patients. A selection of EpCAM-related immune genes were curated from the TISIDB database. To build our model, both univariate and multivariate Cox regression analyses were employed, which enabled us to facilitate the discernment of the pivotal genes worthy of incorporation into study. EpCAM immune-related model were further validated and compared with published models. Association between EpCAM and immune cells was analyzed by ssGSEA, CIBERSORT. Furthermore, an investigation into the relationship between EpCAM expression and immune checkpoints was undertaken. The correlation between EpCAM and therapeutic drugs was analyzed with GSDC database data. The biological function of EpCAM in KIRC was further verified in vitro.

**Fig. 2:**
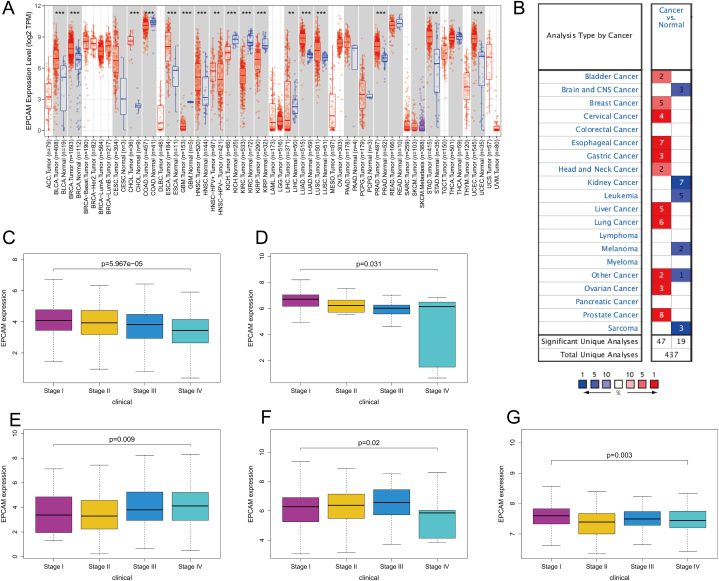
EpCAM expression across a spectrum of cancer types. (A, B) EpCAM expression of pan-cancer. (C, G) EpCAM expression in different stages of KIRC, KICH, HNSC, LUSC, and THCA. Statistical test was the Kolmogorov–Smirnov test.

The content of this study was summarized in Fig. 1. In the TIMER database, the analysis revealed that EpCAM expression exhibited a noteworthy increase in various tumor types when compared to normal tissues. Notably, elevated EpCAM expression was observed in most tumors. Conversely, a significant reduction in EpCAM expression was noted in colon adenocarcinoma (COAD), glioblastoma multiforme (GBM), as well as in liver hepatocellular carcinoma (LIHC) and renal cancer (Fig. 2A). In the Oncomine database, EpCAM expression was significantly higher in BLCA, breast cancer, cervical cancer, ESCA, STAD, HNSC, LIHC, lung cancer, ovarian cancer, and prostate adenocarcinoma (PRAD) but significantly lower in brain cancer, kidney cancer, leukemia, melanoma, and sarcoma than in normal tissues (Fig. 2B). The EpCAM expression in different stages of thirty-three cancers was analyzed, and variances were found only in EpCAM expression in different stages of KIRC, KICH, HNSC, LUSC, and thyroid carcinoma (THCA), as shown in Fig. 2C–G.

### 3.2 Prognosis of EpCAM in pan-cancer

**Fig. 3:**
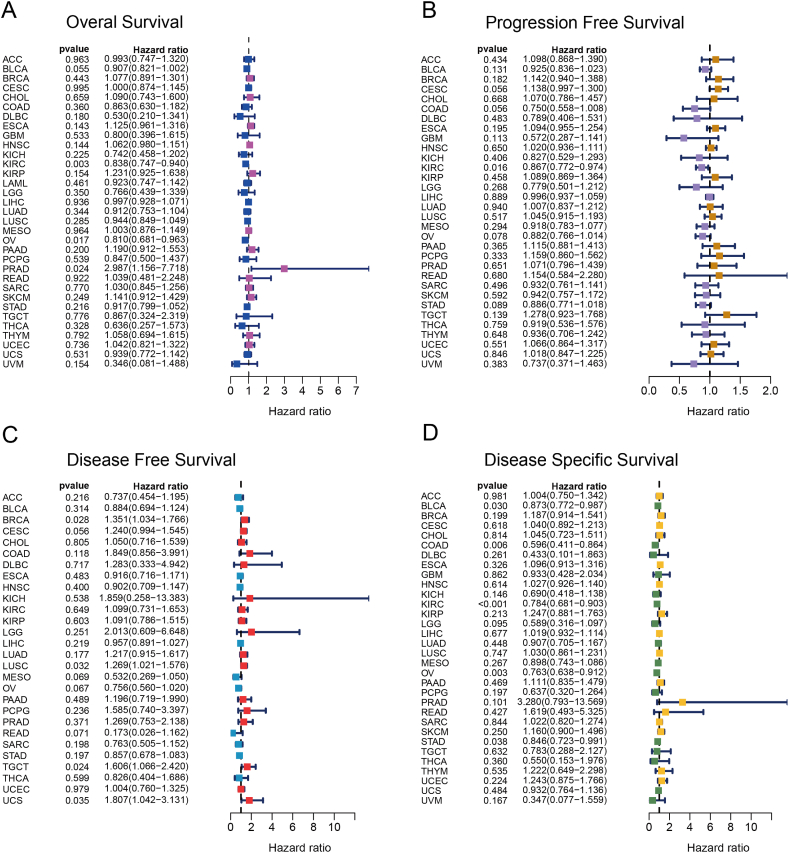
The prognostic utility of EpCAM across various cancer types. (A–D) Individual univariate Cox regression analyses were conducted to evaluate the associations between EpCAM and various survival metrics across a comprehensive range of cancer types.

**Fig. 4:**
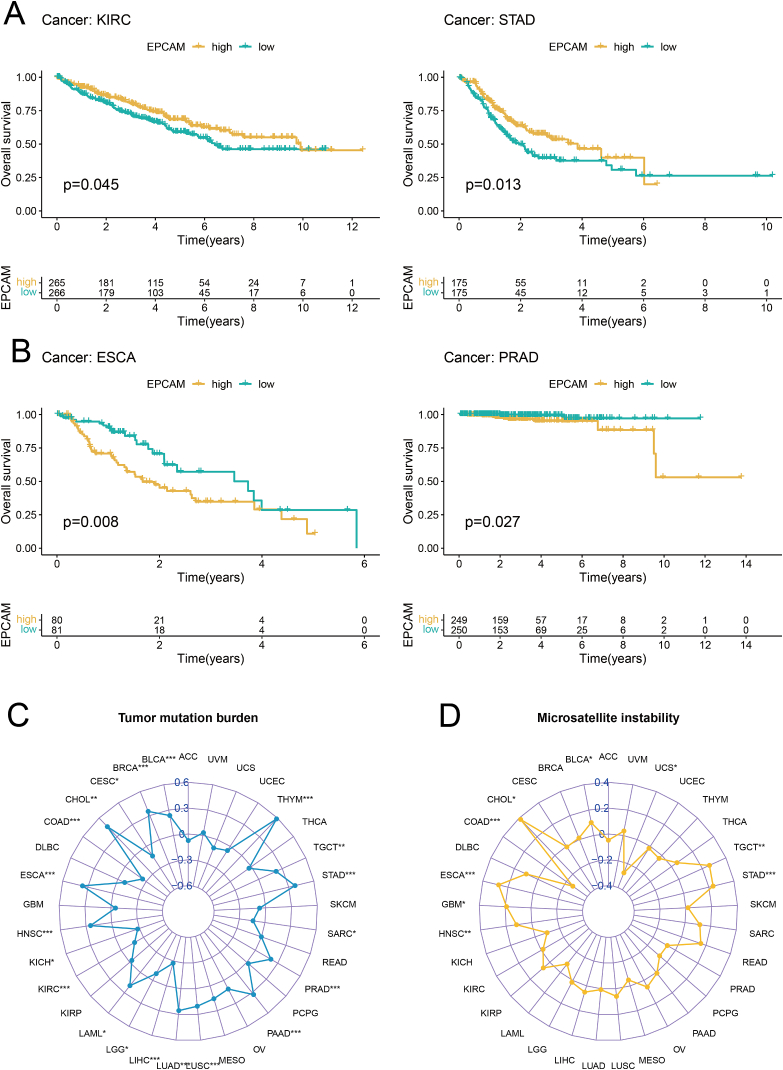
Effect of EpCAM on prognosis and immunity of pan-cancer. (A, B) Kaplan-Meier survival plots were generated for individuals with elevated and diminished EpCAM expression in KIRC, STAD, ESCA, and PRAD. (C, D) Investigation of the association between EpCAM and tumor mutational burden as well as microsatellite instability across a broad spectrum of cancer types. Statistical test was the Spearman correlation test.

To evaluate the prognostic significance of EpCAM in various cancer types, we conducted univariate Cox regression analysis. The results of our analysis demonstrated a noteworthy correlation between the expression of EpCAM and the overall survival (OS) of patients diagnosed with KIRC, OV, and PRAD (Fig. 3A); progression-free survival in KIRC (Fig. 3B); disease-free survival in BRCA, LUSC, TGCT, and UCS (Fig. 3C); and disease-specific survival in BLCA, COAD, KIRC, OV, and STAD (Fig. 3D). K–M survival analysis revealed the upregulation of EpCAM was associated with good prognosis of KIRC and STAD, and poor prognosis of ESCA and PRAD (Fig. 4A and B). Furthermore, EpCAM exhibited a notable correlation with the tumor mutational burden across a wide range of cancer types, including KIRC (Fig. 4C). Additionally, EpCAM's association extended to microsatellite instability, with significant correlations observed in UCS, TGCT, STAD, HNSC, GBM, ESCA, COAD, CHOL, and BLCA (Fig. 4D). The expression, stage, and prognosis of EpCAM in KIRC were significant and related to immunity, so it was selected for further analysis and validation in our own clinical samples.

### 3.3 Evaluation of EpCAM expression and its prognostic relevance in clinical samples of KIRC

**Fig. 5:**
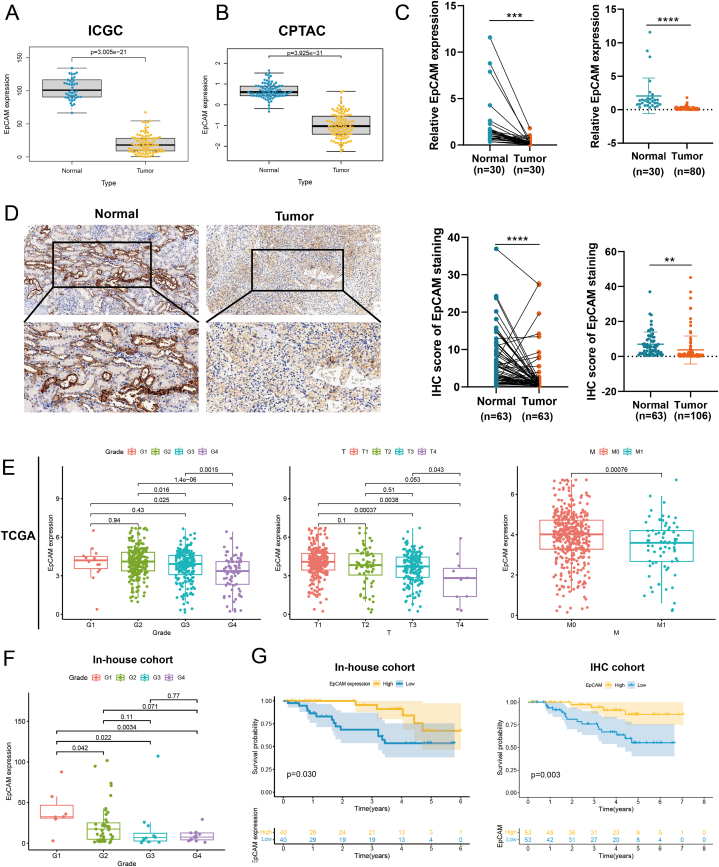
Validation of EpCAM in clinical samples. (A, B) Evaluation of EpCAM expression disparities between non-cancerous and tumor tissues within the ICGA and CPTAC datasets pertaining to KIRC. Statistical tests was the Wilcox test. (C) mRNA expression of EpCAM in 30 paired and unpaired clinical KIRC samples by qRT-PCR. Statistical tests were the paired and unpaired t-tests. (D) Representative IHC images and quantification of EpCAM protein expression in both cancerous and healthy tissues of KIRC. Statistical tests were the paired and unpaired t-tests. (E) EpCAM expression levels across distinct clinical parameters. Statistical test was the Kolmogorov–Smirnov test. (F) EpCAM expression levels among various tumor grades within our in-house cohort. Statistical test was the Kolmogorov–Smirnov test. (G) Survival analyses were performed to assess the prognostic disparities between patients exhibiting heightened and reduced EpCAM expression levels in both our in-house and IHC cohorts.

Within the ICGA and CPTAC databases, a notable reduction in both EpCAM mRNA and protein levels was observed when comparing KIRC to the corresponding normal tissue counterparts (Fig. 5A and B). Further verification by qRT-PCR in clinical samples showed the mRNA expression of EpCAM in cancer tissues was reduced than that in normal tissues in paired and unpaired samples (Fig. 5C). Immunohistochemical analysis of 106 KIRC patients’ samples revealed a substantial reduction in EpCAM protein levels within both paired and unpaired cancer tissues in comparison to normal tissues (Fig. 5D).

In the TCGA dataset, EpCAM was closely related to grade, T, M, and stage (Supplementary Table S2). As grade and T decreased, EpCAM expression increased. Compared with patients with metastasis, EpCAM expression increased in patients without metastasis (Fig. 5E). In the in-house cohort, EpCAM was closely related to grade (Table 1), and as grade increased, EpCAM expression decreased (Fig. 5F).

**Table 1:** Investigation of the association between the expression of EpCAM and clinicopathological characteristics among individuals diagnosed with KIRC within our in-house cohort.

Characteristics	EpCAM expression		Chi-square		*P*
	Low level	High level			
n	36	35			
Grade					
G1+G2	19 (52.8 %)	28 (80.0 %)	5.877		0.015
G3+G4	17 (47.2 %)	7 (20.0 %)			
Age					
≤60	16 (44.4 %)	22 (62.9 %)	2.419		
>60	20 (55.6 %)	13 (37.1 %)			0.120
Sex					
Female	6 (16.7 %)	10 (28.6 %)	1.441		0.230
Male	30 (83.3 %)	25 (71.4 %)			
T					
T1+T2	23 (63.9 %)	20 (57.1 %)	0.338		0.561
T3+T4	13 (36.1 %)	15 (42.9 %)			
N					
N0	27 (75.0 %)	30 (85.7 %)	1.287		0.257
N1	9 (25.0 %)	5 (14.3 %)			
M					
M0	30 (83.3 %)	32 (91.4 %)	1.051		0.305
M1	6 (16.7 %)	3 (8.6 %)			

In the in-house and IHC cohorts, patients exhibiting elevated EpCAM expression, as indicated by both RNA and protein levels, demonstrated a more favorable prognosis in comparison to those with diminished EpCAM expression (Fig. 5G), consistent with the prognosis of TCGA. In the in-house cohort, grade, T, N, and M were unfavorable prognostic factors for patients with KIRC, whereas EpCAM was a favorable prognostic factor for patients (Table 2). In the multivariate Cox regression analysis, EpCAM was identified as a standalone predictor for patient prognosis. Furthermore, both the TCGA dataset and the IHC cohort exhibited adverse prognostic associations of T and M in KIRC patients, as indicated by the univariate Cox regression analysis. Conversely, it underscored EpCAM as a promising and favorable prognostic determinant for patients. (Supplementary Table S3, Table 3). These findings suggest that EpCAM has the potential to emerge as a standalone prognostic marker for KIRC.

**Table 2:** Cox regression examinations of the overall survival of KIRC patients within our in-house cohort.

Clinical features	Univariate analysis			Multivariate analysis		
	HR	95 % CI	*P*	HR	95 % CI	*P*
EpCAM (low/high)	0.688	0.497–0.952	0.024	0.666	0.451–0.985	0.042
Age (≤60 vs > 60)	2.172	0.814–5.693	0.121	1.219	0.395–3.765	0.730
Grade (G1+2/G3+4)	5.002	1.866–13.404	0.001	2.571	0.708–9.339	0.151
T (T1+T2/T3+T4)	3.504	1.337–9.186	0.011	1.845	0.331–10.278	0.485
N (N0/N1)	4.481	1.752–11.457	0.002	0.783	0.121–5.093	0.798
M (M0/M1)	5.933	2.287–15.390	0.000	3.221	0.913–11.360	0.069

**Table 3:** Cox regression examinations of the overall survival of KIRC patients within our IHC cohort.

Clinical features	Univariate analysis			Multivariate analysis		
	HR	95 % CI	*P*	HR	95 % CI	*P*
EpCAM (low/high)	0.339	0.125–0.917	0.033	0.180	0.046–0.701	0.013
Age (≤60 vs > 60)	2.511	0.942–6.693	0.066	2.033	0.619–6.677	0.242
Sex (female/male)	2.414	0.554–10.515	0.240	1.964	0.385–10.008	0.417
Grade (G1+2/G3+4)	1.832	0.722–4.647	0.202	0.689	0.162–2.933	0.614
T (T1+2 vs T3+4)	3.155	1.238–8.043	0.016	1.320	0.257–6.782	0.740
N (N0 vs N1)	6.330	2.408–16.636	0.000	1.328	0.138–12.816	0.806
M (M0 vs M1)	7.325	2.596–20.670	0.000	10.960	1.740–69.042	0.011

### 3.4 Infiltration of immune cell populations in patient groups stratified by varying levels of EpCAM expression

**Fig. 6:**
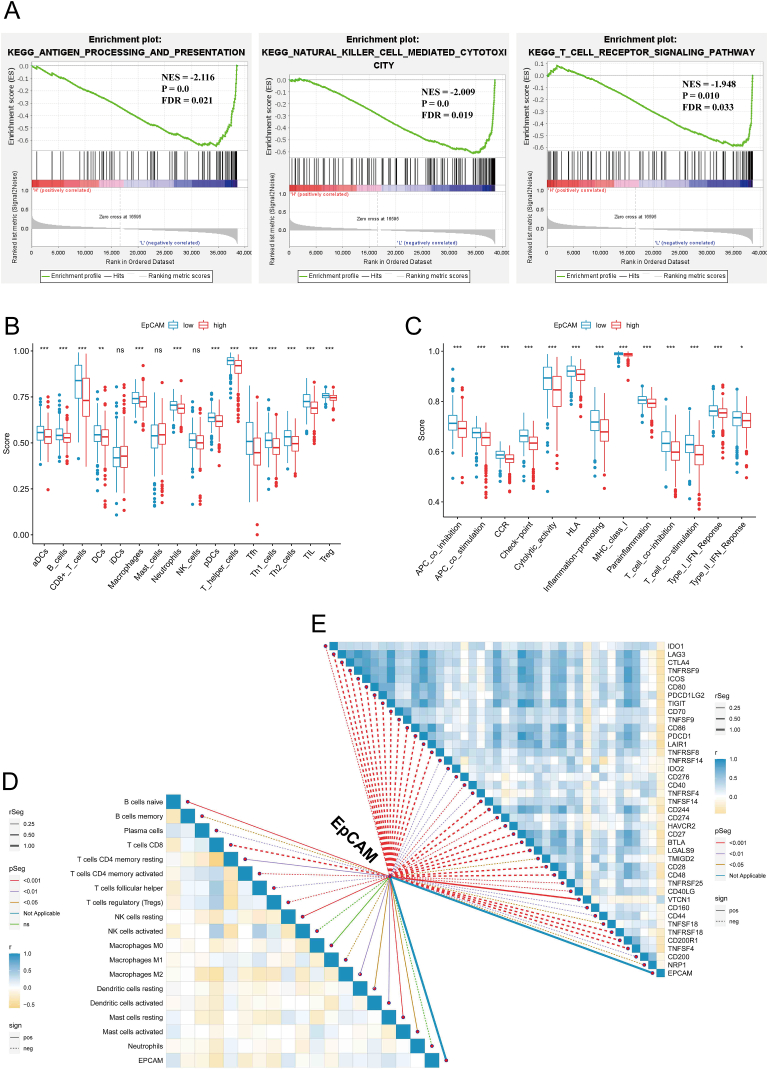
Examination of the relationship between EpCAM expression and infiltration of immune cells. (A) The low EpCAM expression group exhibited a marked enrichment of key immunological processes. (B, C) An investigation was conducted utilizing ssGSEA to assess the immune cell scores and immune functions in two distinct groups classified by their EpCAM expression levels. (D) Examination of immune cell populations within KIRC using the CIBERSORT algorithm was conducted. The statistical assessment employed the Pearson correlation test. (E) An investigation was carried out to establish a correlation between immune checkpoint molecules and the expression of EpCAM. Statistical test was the pearson correlation test.

In order to discern the regulatory modalities and the fundamental mechanisms of EpCAM in the context of KIRC, patients were categorized into high and low EpCAM expression subgroups, with the median expression level serving as the demarcation point. The result reveals substantial enrichment of critical pathways, including antigen processing and presentation, natural killer-cell-mediated cytotoxicity, and T cell receptor signaling pathway, within the low EpCAM expression group (Fig. 6A, Supplementary Table S4). This finding suggests EpCAM may be closely related to the immune function of KIRC. Then, we utilized diverse computational algorithms to delve into the intricate relationship between EpCAM and immune cell populations. The ssGSEA analysis unveiled significant variations in the scores of various immune cell subtypes when comparing the high and low EpCAM expression cohorts. Most immune cells exhibited lower scores in the high EpCAM expression group (Fig. 6B). The analysis revealed a significant reduction in scores across multiple immune regulatory pathways within the high EpCAM expression group. Specifically, APC coinhibition, APC costimulation, CCR, checkpoint, cytolytic activity, HLA, inflammation promotion, MHC class I presentation, para-inflammation, T cell coinhibition, T cell costimulation, type I II IFN response demonstrated decreased scores (Fig. 6C). Utilizing the CIBERSORT algorithm, we conducted a comprehensive analysis of immune cell proportions in individual samples. Our analysis unveiled noteworthy correlations between EpCAM expression and distinct immune cell subpopulations. EpCAM displayed an inverse association with memory B cells, plasma cells, CD8^+^ T cells, activated CD4 memory T cells, Tfh cells, Tregs, activated NK cells, M1 macrophages, and neutrophils (Fig. 6D).

### 3.5 The impact of EpCAM expression upon immune checkpoint

Immunotherapy stands as a cornerstone in contemporary clinical oncology, with extensive and well-developed research dedicated to immune checkpoint inhibitors. In the scope of this study, we delved into the intricate interplay between EpCAM expression and immune checkpoints. Our investigation unveils a prevailing inverse association between the levels of EpCAM expression and the vast spectrum of immune checkpoints (Fig. 6E). The result suggests immune checkpoint inhibitors may exert better therapeutic effects on patients with low EpCAM expression.

### 3.6 Analysis of correlation between EpCAM and therapeutic drugs

Utilizing data sourced from the GSDC database, an assessment was conducted to elucidate the relationship between EpCAM expression and drug IC50 values. The results unveiled distinct correlations. Specifically, the IC50 values of sunitinib, imatinib, and crizotinib exhibited a positive association with EpCAM expression. Conversely, the IC50 value of rapamycin demonstrated a negative correlation with EpCAM expression (Table 4).

**Table 4:** Analysis of anticancer drugs associated with EpCAM.

Gene	Drug name	Cor	Label	*P* value
EpCAM	Sunitinib	0.363	Positive	0.015
EpCAM	Imatinib	0.42	Positive	0.006
EpCAM	Crizotinib	0.307	Positive	0.034
EpCAM	Rapamycin	−0.588	Negative	0.003

EpCAM, epithelial cell adhesion molecule.

### 3.7 Construction of the EpCAM immune-related model

**Fig. 7:**
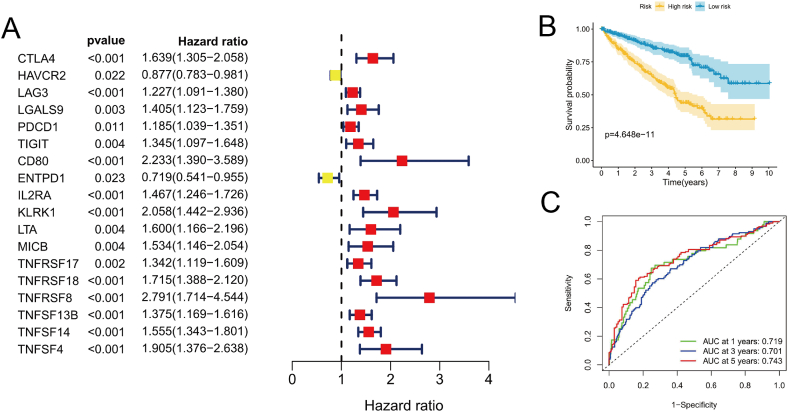
Development of an EpCAM-associated immune model. (A) The identification of predictive genes was accomplished via the application of univariate Cox regression analysis. (B) An examination was carried out to assess the disparity in overall survival between individuals classified as high-risk and those deemed low-risk within the TCGA database, employing the Kaplan-Meier survival curve. The statistical evaluation employed the Log-rank test for significance determination. (C) Evaluation of OS prediction accuracy through ROC curves in KIRC patients.

A stringent screening strategy was implemented with criteria set at R > 0.2 and *P* < 0.05. This led to the identification of thirty-five immunomodulators, which comprised twelve immunoinhibitors and twenty-three immunostimulators. In the subsequent analysis, an initial univariate Cox regression examination pinpointed 18 genes demonstrating significant prognostic potential (Fig. 7A). Following this, a comprehensive multivariate Cox regression analysis successfully winnowed down this selection to a subset of 8 genes, which were subsequently utilized in constructing the prognostic model (Table 5). Risk score = (−0.186 × HAVCR2) + (0.388 × LAG3) + (−0.461 × PDCD1) + (−0.496 × ENTPD1) + (0.335 × IL2RA) + (0.222 × TNFRSF18) + (0.284 × TNFSF14) + (0.354 × TNFSF4). Patients categorized in the high-risk group exhibited a notably reduced OS duration when contrasted with their counterparts in the low-risk group (*P* = 4.648e−11, Fig. 7B). Our model demonstrated robust predictive accuracy, with the areas under the curve (AUC) at 1, 3, and 5 years attaining values of 0.719, 0.701, and 0.743, respectively (Fig. 7C).

**Table 5:** Prognostic immune-related genes for model construction.

Gene	Coef	HR	Lower 95 % CI	Upper 95 % CI
HAVCR2	−0.186	0.830	0.720	0.956
LAG3	0.388	1.475	1.046	2.079
PDCD1	−0.461	0.630	0.432	0.921
ENTPD1	−0.496	0.609	0.445	0.834
IL2RA	0.335	1.398	1.160	1.684
TNFRSF18	0.222	1.248	0.926	1.683
TNFSF14	0.284	1.328	1.128	1.563
TNFSF4	0.354	1.425	0.948	2.142

### 3.8 Validation and comparative assessment of the EpCAM-associated immune model

**Fig. 8:**
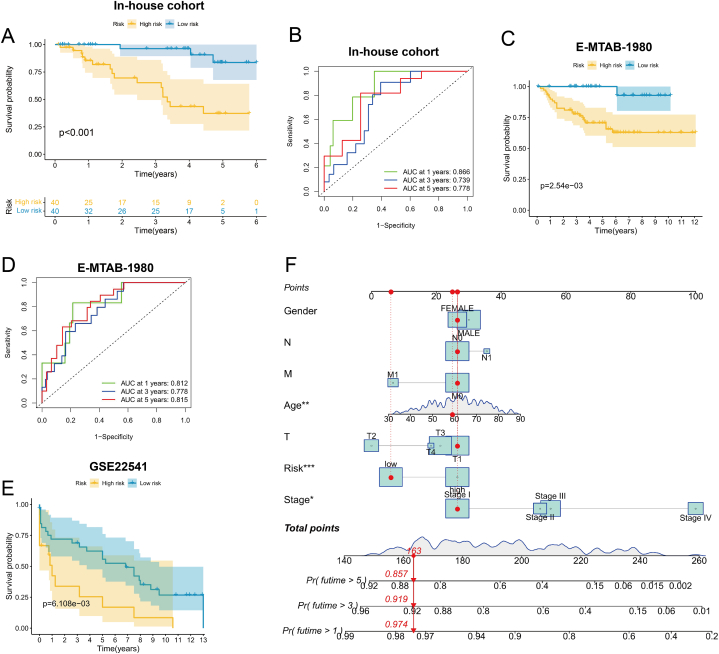
EpCAM immune-related model validation. (A) A Kaplan-Meier survival analysis was executed on an in-house cohort, where they were stratified into distinct high-risk and low-risk groups (n = 80). The statistical assessment was carried out using the Log-rank test. (B) ROC curves were employed to gauge the predictive efficacy of the EpCAM immune-related model within our in-house cohort (n = 80). (C) Overall survival analysis in the E-MTAB-1980 dataset (n = 101). Log-rank test was used for statistical comparison. (D) ROC curves were constructed to evaluate the predictive accuracy of the model in the E-MTAB-1980 dataset (n = 101). (E) Overall Survival analysis in the GSE22541 Dataset. Statistical assessment was performed using the Log-rank test. (F) A nomogram was developed based on scores derived from the EpCAM immune-related model and relevant clinicopathological variables.

In order to assess the model's validity, we conducted external validation in both our in-house cohort and publicly available datasets. In both our in-house cohort and the E-MTAB-1980 dataset, individuals classified as high-risk demonstrated an inferior prognosis in contrast to their low-risk counterparts. Notably, the ROC curves consistently showed AUC values surpassing 0.7 (Fig. 8A–D). Likewise, in the GSE22541 dataset, we detected marked disparities in survival outcomes between patient groups classified as high-risk and low-risk (*P* = 6.108e−03, Fig. 8E). To assess the practical clinical utility of our model, we designed a nomogram that amalgamates risk scores with clinicopathological data for the prognosis prediction of KIRC patients at 1, 3, and 5 years (Fig. 8F). Furthermore, we undertook a thorough review of 137 previously published models and determined that our model's C-index surpassed those of other models (Supplementary Fig. S1).

### 3.9 The involvement of EpCAM in the biological mechanisms of KIRC

**Fig. 9:**
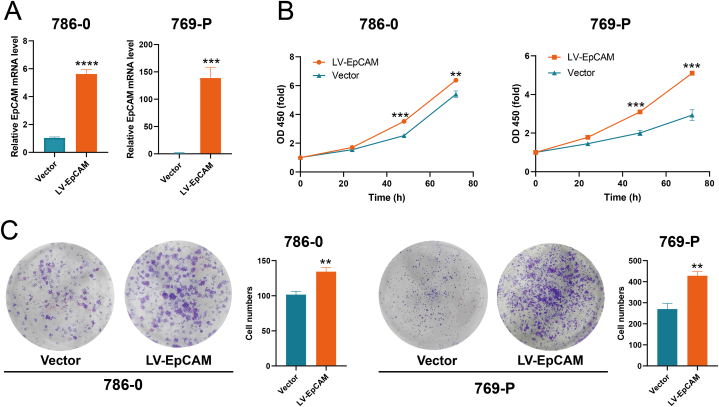
The effect of EpCAM on proliferation in KIRC. (A) Detection of overexpression efficiency by qRT-PCR (n = 3). (B) OD value changes after EpCAM overexpression (n = 3). (C) Effect of overexpression of EpCAM on cell colony formation ability (n = 3). Statistical evaluation for all measurements was performed through the utilization of the Student's t-test.

**Fig. 10:**
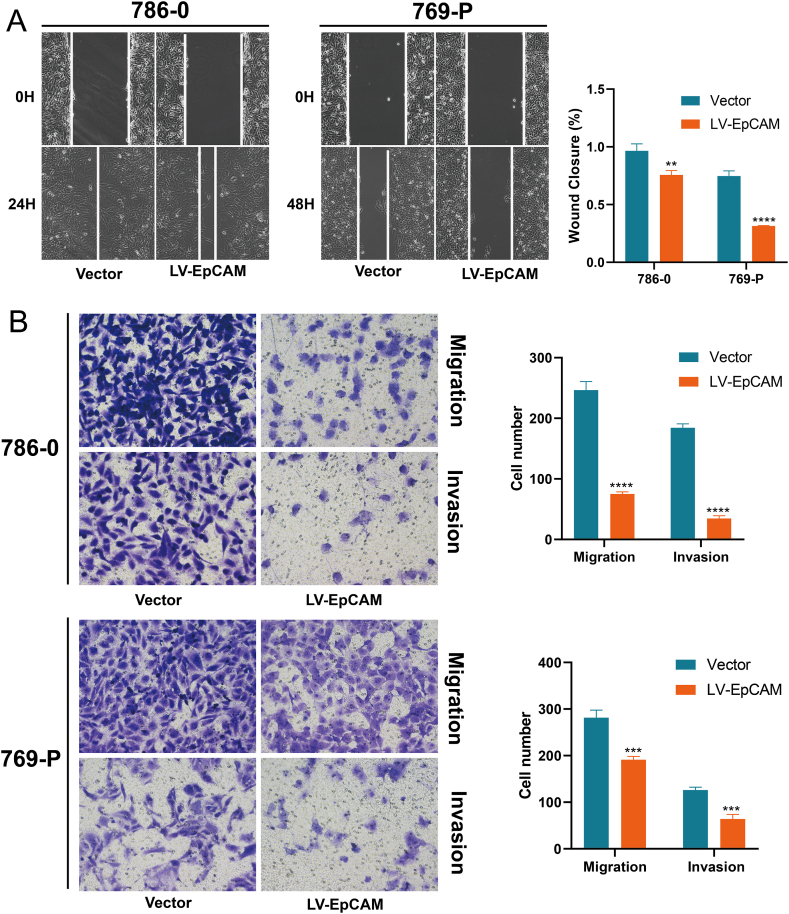
Effect of EpCAM on KIRC progression. (A) Wound healing assay for cell migration inhibition. (B) EpCAM overexpression significantly inhibited the tumor cells migration and invasion abilities by the transwell assay (n = 3). Statistical evaluation for all measurements was performed through the utilization of the Student's t-test.

Following lentiviral overexpression in 786-0 and 769-P cells, qRT-PCR was used to detect the overexpression effect, and the results revealed EpCAM expression could be significantly increased (Fig. 9A). The observed overexpression efficiency reached approximately 130 times compared to the negative control group. This substantial increase was attributed to the initially low expression of EpCAM in 769-P cells. The introduction of EpCAM overexpression prominently elevated cell proliferation capacity, as indicated by the CCK-8 assay and colony formation assay (Fig. 9B and C). Conversely, in the wound healing assay, we observed that EpCAM overexpression impeded cell migration (Fig. 10A). These findings were substantiated by the results of the transwell assay, which not only confirmed the reduction in the migratory potential of tumor cells post-EpCAM overexpression but also demonstrated a substantial inhibition in invasion ability (Fig. 10B). These results indicate EpCAM had dual effects of promoting proliferation and preventing metastasis in KIRC.

## 4 Discussion

Metastasis stands as a crucial biological hallmark of malignant tumors, with CAMs assuming a pivotal role in driving this intricate process. EpCAM is the earliest biomarker found in CAMs. EpCAM exhibits carcinogenic effects in many tumors and participates in malignant progression [[19], [20], [21], [22], [23], [24], [25]]. EpCAM is an unfavorable prognostic factor, and its overexpression can promote metastasis in nasopharyngeal carcinoma (NPC) [26]. Tumor heterogeneity leads to distinct functions of EpCAM across various tumor types. Low expression of EpCAM can reduce the possibility of cancer cell progression [27]. The expression of EpCAM is dynamic, the function is context dependent, and different functions are achieved through cellular signaling [28,29]. The function of EpCAM in KIRC remains to be definitively elucidated.

A pan-cancer analysis holds the potential to discern variances among distinct cancer types, enabling tailored therapeutic approaches for individual cancer patients. First, the expression and prognosis of EpCAM in thirty-three types of tumors was analyzed. Consistent with previous reports [[30], [31], [32], [33], [34], [35]], EpCAM exhibited significant overexpression in the majority of cancer types. The expression, stage, and prognosis of EpCAM in KIRC were significant and related to immunity, so it was selected for further analysis and validation. Our findings were in accordance with the database results, demonstrating a notable decline EpCAM expression within cancer tissues in our clinical specimens. EpCAM expression increased in patients with low grade and without metastasis. Christian et al. [36] only found that EpCAM protein levels can function as independent prognostic markers for patients with KIRC, without mRNA levels. However, EpCAM mRNA and protein levels were both used as independent, favorable prognostic factors in patients with KIRC in the public data set and in our own cohort. Our research was consistent with the consensus that distant metastasis stands as the primary factor contributing to mortality among individuals diagnosed with KIRC.

Low EpCAM expression was closely related to immunity. The study demonstrated that heightened EpCAM expression exerts suppressive effects on the cytotoxic function executed by NK cells [37]. Irrespective of the computational approaches, including ssGSEA or CIBERSORT algorithms, our findings consistently demonstrated an inverse association between EpCAM expression and the abundance of various immune cell types, including CD8^+^T cells and Tregs. It is noteworthy that a heightened presence of CD8^+^T cells exhibited an association with a less favorable prognosis among KIRC patients [38]. Although immune cell infiltration in patients with low EpCAM expression was extensive, EpCAM was negatively correlated with most immune checkpoints, likely because the overexpression or strong function of immune checkpoints could inhibit the immune function of patients with low EpCAM expression. Our investigation indicates that patients with diminished EpCAM expression may experience enhanced therapeutic efficacy when treated with immune checkpoint inhibitors, ultimately bolstering the immune response against the tumor. IL-8 has the capacity to enhance the expression of EpCAM in ovarian cancer cells, while conversely, IL-6 exerts an inhibitory effect on this specific phenomenon [39]. IFN-γ was highly expressed in liver NK cells, which can promote the progression of liver cancer through the EpCAM–EMT axis [40]. In light of EpCAM's significant involvement in tumor immunity, we developed a prognostic model incorporating eight immune-related genes. Our model demonstrated commendable predictive accuracy, translating into a more favorable prognosis for patients categorized as low-risk, in contrast to their high-risk counterparts. The validation of our model was conducted within our in-house cohort, alongside the E-MTAB-1980 and GSE22541 datasets, collectively substantiating its robust utility. Furthermore, the integration of risk scores and clinicopathological variables into nomograms demonstrated proficient prognostic capacity, facilitating the accurate prediction of OS for KIRC patients and thereby holding potential for clinical implementation.

Research investigations have revealed a strong association between EpCAM and drug resistance across multiple cancer types, including ovarian cancer [41], breast cancer [11], and leukemia [42]. In our study, patients with high EpCAM expression showed increased resistance to sunitinib, imatinib, and kezotinib and increased sensitivity to rapamycin. Sunitinib is commonly used in patients with clinical KIRC, but some patients had drug resistance and the therapeutic effect was not obvious. Many genes have been found involved in KIRC sunitinib resistance [[43], [44], [45], [46], [47], [48]]. EpCAM can also be used as a drug resistance indicator and guide the clinical medication of patients with KIRC.

In our study, EpCAM had the dual effects of promoting proliferation and preventing metastasis, which provided new insights for EpCAM in KIRC. Wen et al. [49]found high expression of EpCAM was beneficial for the prognosis of endometrial cancer, and knocking down EpCAM can promote cancer cell invasion and reduce proliferation, which was consistent with our findings. FBXO22 exhibited a dual function in breast cancer, enhancing cell proliferation while concurrently suppressing metastatic processes [50]. Epigenetic regulation led to inconsistencies in the growth and mobility capabilities of cancerous cells [51]. Combined with our results, EpCAM may promote the formation of tumor colonies by enhancing tumor cell activity in the early stage of tumor formation. When metastatic tumors develop, EpCAM may promote the migration and invasion of tumor cells by inhibiting its expression through some mechanism. Our investigation indicated that targeting EpCAM may not be a viable therapeutic approach for KIRC. However, our study did have certain constraints that warrant consideration. Firstly, additional research is essential to elucidate the precise mechanisms underlying the suppression of EpCAM expression in metastatic tumors, as it relates to the promotion of tumor migration and invasion. Secondly, there is a need for experimental exploration of EpCAM's involvement in immune-related processes.

## 5 Conclusions

We constructed a robust immune-related model. Importantly, this paper revealed EpCAM has the dual effects of promoting proliferation and preventing metastasis in KIRC.

## Data availability statement

Data included in article/supplementary material/referenced in article.

## CRediT authorship contribution statement

**Mei Chen:** Writing – original draft, Software, Conceptualization. **Yuanhui Gao:** Resources, Methodology, Investigation, Formal analysis. **Hui Cao:** Visualization, Validation, Supervision, Project administration. **Zhenting Wang:** Writing – review & editing, Supervision, Resources, Data curation. **Shufang Zhang:** Writing – review & editing, Investigation, Funding acquisition, Formal analysis, Data curation.

## Declaration of competing interest

The authors declare that they have no known competing financial interests or personal relationships that could have appeared to influence the work reported in this paper.
