# Supplementary material for: Comprehensive analysis reveals dual biological function roles of EpCAM in kidney renal clear cell carcinoma
Source: Heliyon. 2023 Dec 14;10(1):e23505. doi: 10.1016/j.heliyon.2023.e23505 (PMC10767389; doi:10.1016/j.heliyon.2023.e23505)
Supplement: Multimedia component 1 [file mmc1.docx]

Supplementary Table 1. Primers used in the study

| Gene | Forward primer (5’ to 3’) | Reverse primer (5’ to 3’) |
| --- | --- | --- |
| EpCAM | GTCTGTGAAAACTACAAGCTGG | CAGTATTTTGTGCACCAACTGA |
| GAPDH | CAGGAGGCATTGCTGATGAT | GAAGGCTGGGGCTCATTT |
