# Supplementary material for: Comprehensive analysis reveals dual biological function roles of EpCAM in kidney renal clear cell carcinoma
Source: Heliyon. 2023 Dec 14;10(1):e23505. doi: 10.1016/j.heliyon.2023.e23505 (PMC10767389; doi:10.1016/j.heliyon.2023.e23505)
Supplement: Multimedia component 2 [file mmc2.docx]

Correlation analysis between EpCAM expression and clinicopathological features in KIRC patients from the TCGA database

| Characteristics | EpCAM expression | | Chi-square | *P* |
| --- | --- | --- | --- | --- |
|  | Low level | High level |  |  |
| n | 245 | 244 |  |  |
| Grade |  |  |  |  |
| G1+G2 | 95 (38.8%) | 126 (51.6%) | 8.167 | 0.004 |
| G3+G4 | 150 (61.2%) | 118 (48.4%) |  |  |
| Age |  |  |  |  |
| <=60 | 122 (49.8%) | 121 (49.6%) | 0.002 | 0.964 |
| >60 | 123 (50.2%) | 123 (50.4%) |  |  |
| Gender |  |  |  |  |
| Female | 80 (32.7%) | 86 (35.2%) | 0.367 | 0.545 |
| Male | 165 (67.3%) | 158 (64.8%) |  |  |
| T |  |  |  |  |
| T1+2 | 140 (57.1%) | 166 (68.0%) | 6.191 | 0.013 |
| T3+4 | 105 (42.9%) | 78 (32.0%) |  |  |
| M |  |  |  |  |
| M0 | 196 (80.0%) | 216 (88.5%) | 6.696 | 0.010 |
| M1 | 49 (20.0%) | 28 (11.5%) |  |  |
| Stage |  |  |  |  |
| Ⅰ+Ⅱ | 131 (53.5%) | 158 (64.8%) | 6.440 | 0.011 |
| Ⅲ+Ⅳ | 114 (46.5%) | 86 (35.2%) |  |  |
