# Supplementary material for: Comprehensive analysis reveals dual biological function roles of EpCAM in kidney renal clear cell carcinoma
Source: Heliyon. 2023 Dec 14;10(1):e23505. doi: 10.1016/j.heliyon.2023.e23505 (PMC10767389; doi:10.1016/j.heliyon.2023.e23505)
Supplement: Multimedia component 3 [file mmc3.docx]

Supplementary Table 3 Univariate and multivariate Cox regression analyses of overall survival in KIRC patients from the TCGA database

| Characteristics | Univariate analysis | | | Multivariate analysis | | |
| --- | --- | --- | --- | --- | --- | --- |
|  | HR | 95% CI | *P* | HR | 95% CI | *P* |
| EpCAM (low/high) | 0.850 | 0.756-0.956 | 0.007 | 0.880 | 0.776-0.997 | 0.045 |
| Age (<=60 vs >60) | 1.768 | 1.287-2.428 | 0.000 | 1.649 | 1.197-2.273 | 0.002 |
| Gender (Female/male) | 0.920 | 0.669-1.266 | 0.610 | 0.928 | 0.668-1.290 | 0.658 |
| Stage (Ⅰ+Ⅱ vs Ⅲ+Ⅳ) | 4.110 | 2.951-5.724 | 6.11E-17 | 2.401 | 1.159-4.975 | 0.018 |
| T (T1+2 vs T3+4) | 3.397 | 2.475-4.663 | 3.79E-14 | 1.103 | 0.586-2.074 | 0.761 |
| M (M0 vs M1) | 4.304 | 3.126-5.933 | 4.94E-19 | 2.368 | 1.602-3.501 | 1.53E-05 |
