# Supplementary material for: Comprehensive analysis reveals dual biological function roles of EpCAM in kidney renal clear cell carcinoma
Source: Heliyon. 2023 Dec 14;10(1):e23505. doi: 10.1016/j.heliyon.2023.e23505 (PMC10767389; doi:10.1016/j.heliyon.2023.e23505)
Supplement: Multimedia component 4 [file mmc4.docx]

Supplementary Table 4 Low EpCAM expression phenotype enriched gene set

| Gene set | ES | NES | NOM p-val | FDR q-val |
| --- | --- | --- | --- | --- |
| Antigen processing and presentation | -0.647 | -2.116 | 0.000 | 0.021 |
| Natural killer cell mediated cytotoxicity | -0.614 | -2.099 | 0.000 | 0.019 |
| T cell receptor signaling pathway | -0.590 | -1.948 | 0.010 | 0.033 |
