# Supplementary figures and images for: Comprehensive analysis reveals dual biological function roles of EpCAM in kidney renal clear cell carcinoma
Source: Heliyon. 2023 Dec 14;10(1):e23505. doi: 10.1016/j.heliyon.2023.e23505 (PMC10767389; doi:10.1016/j.heliyon.2023.e23505)

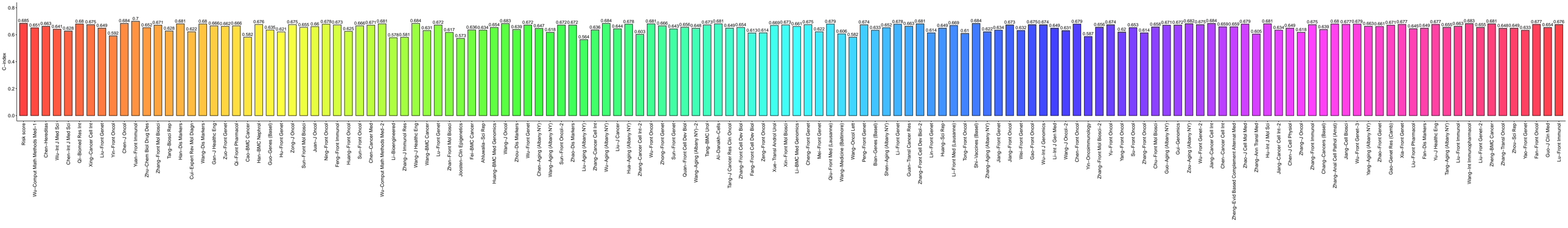

Supplement: Multimedia component 8 [file mmc8.pdf]
